# Grouping facilitates avoidance of parasites by fish

**DOI:** 10.1186/1756-3305-6-301

**Published:** 2013-10-17

**Authors:** Victor N Mikheev, Anna F Pasternak, Jouni Taskinen, Tellervo E Valtonen

**Affiliations:** 1 Laboratory of Behaviour of Lower Vertebrates, Institute of Ecology and Evolution, Russian Academy of Sciences, 33 Leninskii pr, 119071, Moscow, Russia; 2 Laboratory of Plankton Ecology, Institute of Oceanology Russian Academy of Sciences, 36 Nakhimovskii pr, 117997, Moscow, Russia; 3 Department of Biological and Environmental Science, University of Jyväskylä, PL 3540351, Jyväskylä, Finland

**Keywords:** Parasite avoidance, Heterogeneous habitat, *Diplostomum pseudospathaceum*, Rainbow trout, Fish grouping

## Abstract

**Background:**

Parasite distribution is often highly heterogeneous, and intensity of infection depends, among other things, on how well hosts can avoid areas with a high concentration of parasites. We studied the role of fish behaviour in avoiding microhabitats with a high infection risk using *Oncorhynchus mykiss* and cercariae of *Diplostomum pseudospathaceum* as a model. Spatial distribution of parasites in experimental tanks was highly heterogeneous. We hypothesized that fish in groups are better at recognizing a parasitized area and avoiding it than solitary fish.

**Methods:**

Number of fish, either solitary or in groups of 5, was recorded in different compartments of a shuttle tank where fish could make a choice between areas with different risk of being infected. Intensity of infection was assessed and compared with the number of fish recorded in the compartment with parasites and level of fish motility.

**Results:**

Both solitary fish and fish in groups avoided parasitized areas, but fish in groups avoided it more strongly and thus acquired significantly fewer parasites than solitary fish. Prevalence of infection among grouped and solitary fish was 66 and 92 %, respectively, with the mean abundance two times higher in the solitary fish. Between-individual variation in the number of parasites per fish was higher in the “groups” treatment (across all individuals) than in the “solitary” treatment. Avoidance behaviour was more efficient when fish were allowed to explore the experimental arena prior to parasite exposure. High motility of fish was shown to increase the acquisition of *D. pseudospathaceum*.

**Conclusion:**

Fish in groups better avoided parasitized habitat, and acquired significantly fewer parasites than solitary fish. We suggest that fish in groups benefit from information about parasites gained from other members of a group. Grouping behaviour may be an efficient mechanism of parasite avoidance, together with individual behaviour and immune responses of fishes. Avoidance of habitats with a high parasite risk can be an important factor contributing to the evolution and maintenance of grouping behaviour in fish.

## Background

Parasites are known to affect the physiology, behaviour and life history of their hosts, over both evolutionary and ecological scales [1-4], and hosts exhibit various counter-adaptations to deal with parasites [3]. Host behaviour plays an important role in defence against parasites [4,5]. A broad array of host behaviours, such as escape responses, changes in habitat choice and social behaviours, are involved in parasite avoidance [6-8]. Increased parasitism has been considered one of the costs of sociality [9]. However, sociality may also include benefits in terms of defence against parasites, such as lower exposure due to dilution effects when living in groups [10,11].

Fish are able to avoid parasitized habitats where large and easily recognizable parasites such as *Argulus canadensis* occur [12]. Many infective free-swimming parasite stages, like trematode cercariae, are small. Their first intermediate hosts, snails or clams, shed “clouds” of tiny cercariae, which are often very unevenly distributed. This makes detection and avoidance of trematode cercariae difficult. Individual fish may only become aware of the parasite’s presence when irritated by penetrating cercariae [13,14]. Juvenile rainbow trout, *Oncorhynchus mykiss*, experimentally exposed to cercariae of the trematode *Diplostomum spathaceum*, fled from the site of high parasite concentration shortly after penetration of the first few cercariae [8]. However, the persistence of such avoidance behaviour is unclear. One or a few *D. spathaceum* metacercariae established in fish eyes will not cause serious damage [15,16], but as more cercariae are accumulated in a fish, the more deleterious are the effects [15,16]. Fish should thus avoid acquisition of numerous parasites.

Grouping behaviour of fish is well known to be crucial in anti-predator defence, i.e. in avoidance of sites with high predation risk [11,17], but its role in anti-parasite defence is poorly understood [18]. In the presence of *Diplostomum* spp. cercariae, freshwater fish formed tighter shoals than in the absence of these parasites [19]. Shoaling in sticklebacks is an effective mechanism, acting through the dilution effect, to avoid the ectoparasite *Argulus canadensis*[10]. A meta-analysis revealed that the intensity of infection with mobile parasites consistently declined with group size [20]. However, to our knowledge, the role of fish grouping in avoiding areas with high risk of infection has not been studied. Finding such an effect would indicate that fish may be able to obtain information about spatial risk of parasite exposure by observing other members of the shoal. Indeed, learned defence (i.e. social learning) has been shown to take place in fish shoals; for example, information about predation risk is socially transmitted from the observer(s) to the other members of the group [11,21].

We have used an experimental arena (a shuttle tank where fish could make a choice between areas with different levels of risk of being infected) and an experimental fish-parasite model (juvenile *Oncorhynchus mykiss* and *Diplostomum pseudospathaceum*) to study if microhabitat selection could help fish to avoid parasitized areas when the parasite distribution is highly clumped. Our hypothesis is that fish in groups could better recognize and avoid parasitized areas than solitary fish.

## Methods

### Fish and parasites

Fish were obtained from a commercial fish farm. Fish were reared in indoor tanks supplied with ground water. The fish were free of *D. pseudospathaceum* infection. Mean fish fork length ± s.e. was 91.5 ± 8.62 mm (Experiment 1) and 93.8 ± 8.26 mm (Experiment 2). The mean body lengths were not significantly different between 'groups’ and 'solitary fish’ treatments. Prior to the experiments, about 300 fish were kept in a flow-through tank of 2.5 m^3^ on 8:16 L : D cycle at 16°C; they were fed with commercial pelleted food (1.5 mm size, Nutra Parr LB, Norway).

Cercariae of *D. pseudospathaceum* were obtained from 8 naturally infected *Lymnaea stagnalis* snails collected from Lake Konnevesi. *D. pseudospathaceum* is the only diplostomid species found in this snail in Lake Konnevesi [22,23]. We pooled all cercariae produced within 6 hours and estimated their density from ten 1-ml subsamples of the suspension. Infectivity of *D. pseudospathaceum* cercariae does not decrease even 10 hours after shedding at 20°C [24].

### Experimental set-up and procedure

The experiments were conducted at the Konnevesi Research Station, University of Jyväskylä in July – August 2006. Two experiments were conducted to study avoidance of a parasitized area by solitary and grouped fish. In the first experiment, fish were not allowed to explore the experimental arena prior to the beginning of testing. In the second experiment, fish were allowed to explore the experimental arena for 120-min prior to testing. The number of parasites acquired by fish was estimated. Young-of-the-year rainbow trout *Oncorhynchus mykiss* were used for the experiments.

Four flow-through dark brown 3-compartment tanks (total length × width × height 170 × 30 × 40 cm, volume 180 l) were used. The compartments contained no stones or cover and the bottom was flat and plain. The two end compartments (70 × 30 × 40 cm each) were connected to the central one (30 × 30 × 40 cm) by rectangular holes of 5 × 3 cm near the bottom. The holes could be closed and opened with raising doors to control the passage of fish from one compartment to another. The central compartment was used as a start chamber. In trials with parasites, cercariae were added to a randomly chosen end compartment and filtered lake water to the opposite end compartment. In control trials (without parasites), filtered lake water without parasites was added to both end compartments. At the beginning of each test, the water flow was turned off. Oxygen saturation was 90–97% and it did not decrease by more than 1 – 3% by the end of experiment. Water temperature was kept at 15-16°C; illumination was 300 lux. The water was removed and the tanks were thoroughly cleaned and rinsed between the trials.

In our preliminary tests, strong vertical and weak horizontal dispersion of *D. pseudospathaceum* cercariae was observed see also [25]. Cercariae were placed in a 100-ml bottle on the bottom of the aquarium (40 × 30 × 20 cm). After 30 min, a majority of them left the bottle and concentrated in the upper and, to a lesser extent, near-bottom layers in the vicinity (about 5 cm) of the bottle. To check if a difference in cercariae concentration between “parasitized” and “opposite” compartments of the experimental tanks still existed by the end of a 3 h exposure, five 100-ml samples were randomly taken from each compartment. From 2 to 15 cercariae were found in samples from the parasitized compartment, while only once 2 cercariae were found in the whole set of samples from the opposite compartment. This suggests that the substantial difference in cercariae concentration between the end compartments was maintained until the end of the 3 h experiment.

To study if fish avoided a parasitized compartment, the number of fish in the two end compartments and central compartment was recorded every 15 min over 3 hrs (Experiment 1) and over 30 min (Experiment 2). Each recording consisted of 3 consecutive counts with a 1 – min interval between them. The sum of the three counts for each 15 min was used for further analysis. For simplicity, this sum will then be called “number of fish recorded” (NFR, ind). The more time a fish spent in a certain compartment, the higher the probability it would be recorded there, and the higher the obtained NFR would be for this compartment.

### Experiment 1

In this experiment, we studied 1) if fish could avoid a parasitized area and 2) if they did it better when in groups than as solitary fish. Fish were allowed to settle in the central compartment for 15 min, then the doors were gently raised. A suspension of *D. pseudospathaceum* cercariae in filtered lake water was slowly added through a tube (diameter 2.5 mm) into a randomly chosen end compartment. Filtered lake water without parasites was added in the same way to the opposite end compartment. Three hundred millilitres of suspension containing 12,000 cercariae were released over 20 minutes. In control tests, instead of parasite suspension, filtered lake water without parasites was added to both end compartments.

The first recording was done 15 min after the doors were opened. Observers monitored fish from behind a screen through slits. Four control tests and 4 tests where fish were exposed to parasites were run daily from 11 to 16 o’clock. In total, we carried out 32 replicates with solitary fish (16 with parasites, 16 control) and 20 with groups of 5 fish (10 with parasites, 10 control).

### Experiment 2

In this experiment, we studied how solitary fish and fish in groups avoided a parasitized compartment and assessed the number of parasites acquired by each fish. Fish were allowed to get familiar with the experimental arena over two hours before the test started. A bottle of 240 ml lake water containing 12,000 *D. pseudospathaceum* cercariae was placed in the parasitized end compartment. A bottle of 240 ml filtered lake water without parasites was placed in the opposite end compartment.

Each trial lasted for 30 min. We assessed NFR for the parasitized and nonparasitized compartments (24 replicates for solitary fish and 24 replicates for fish in groups of 5). We also assessed the motility of the fish in groups (24 replicates). The number of fish in motion and resting motionless on the bottom was counted at every recording. If 3 or more fish out of 5 were in motion at the recording point, the whole group was scored as of “high motility”. If 3 or more fish of 5 were motionless, the whole group was scored as of “low motility”. For each group of 5 fish, 9 such scores were accumulated by the end of the 30-min observation. If 5 or more of the 9 scores were “high motility”, the group was considered as a “high motility” group.

After exposure, fish were transferred to 150 l flow-through tanks where they were kept for 2 days, which is the time needed for *D. pseudospathaceum* metacercariae to develop in the eye lenses to an easily recognizable size. Then the fish were killed by an overdose of MS222, measured, and inspected for the number of parasites in the eye lenses. The number of established metacercariae was counted microscopically for all the 24 fish in the tests on solitary fish and 120 fish in the tests on groups.

### Data analysis

The NFR values for the end compartments were pooled either for the whole 180-min, or for the first and second 90-min periods separately (Experiment 1), or for the whole 30-min period (Experiment 2), and they were used as response variables in statistical analyses. For comparisons between solitary and grouped fish, the values for the groups of 5 fish were divided by 5.

Two-way ANOVA with effects of parasites (presence or absence) and fish group (solitary *vs* group) was used to compare the NFR values for different compartments of the experimental tanks. The data were checked for normality and homogeneity of variances and met the assumptions of ANOVA. LSD test was used for post-hoc comparisons.

Mann–Whitney U test was used to compare the number of parasites acquired by solitary fish and fish in groups, and by high and low motility fish groups in Experiment 2. Spearman correlation analysis was used to assess the relationship between the number of acquired metacercariae and the NFR for the parasitized compartment. All analyses were conducted with STATISTICA 6.0 software.

### Ethical note

We used 0+ *Oncorhynchus mykiss*. The level of experimental *D. pseudospathaceum* infection was maintained at a much lower level than maximum values reported for naturally occurring infections (up to 200–500 ind fish^-1^) [26,27]. The mortality of infected fish in these experiments was less than 1% and did not exceed that of control fish. No visible damage was observed in any fish. We minimized the required number of animals that were killed and dissected. Experimental fish were killed at the end of the tests with an overdose of MS 222, and dissected. In total, 210 experimentally infected fish were killed. The experiments were conducted with permission of the Lab-Animal Care and Use Committee of the University of Jyväskylä (licence number 30/30.5.2005).

## Results

### Experiment 1

**Figure 1:**
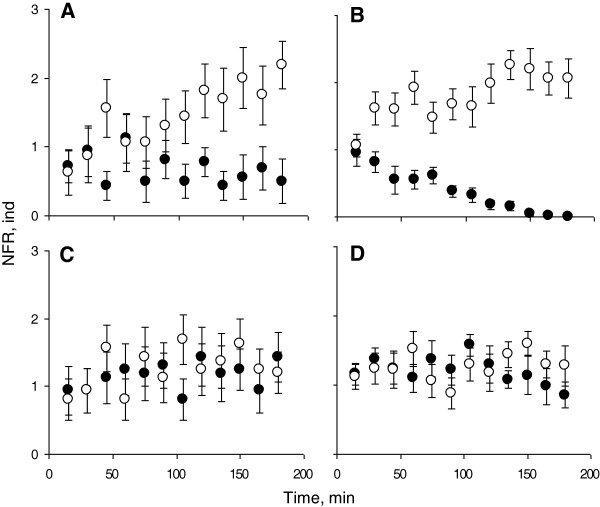
**Distribution of fish between two compartments in Experiment 1. A**, **B**: trials where lake water with parasites was added to one of the end compartments. **C**, **D**: control trials where lake water without parasites was added to both end compartments. **A**, **C** – tests on solitary fish; **B**, **D** – tests on fish in groups of 5. NFR, ind – index of fish number in different compartments as a sum of 3 consecutive counts of fish for each 15 min. NFR values for the groups of 5 fish were divided by 5. Black circles: NFR for the parasitized compartment, or for a randomly chosen compartment in the control trials. Open circles: NFR for the opposite compartment. Means and SE bars are shown.

Both solitary and grouped juveniles of *O. mykiss* avoided the compartment with *D. pseudospathaceum* cercariae. Significantly lower values of the “number of fish recorded”, NFR, were obtained for the parasitized end compartment than for the opposite compartment without parasites (Experiment 1, Figure 1A, B) (Two-way ANOVA, *F*_1,1_ = 31.21; *P* < 0.0001). No difference in the NFR between the end compartments in the control trials was found (Figure 1C, D) (Two-way ANOVA, *F*_1,1_ = 1.63; *P* = 0.209. LSD post-hoc test: *P* = 0.263 for solitary and *P* = 0.506 for grouped fish).

There was no significant difference in avoidance of the parasitized compartment between solitary fish and fish in groups when the NFR values were pooled for the whole 180-min period (Two-way ANOVA, *F*_1,1_ = 0.97; *P* = 0.329; LSD post-hoc test for the NFR in tests on solitary fish *vs* fish in groups: *P* = 0.097) (Figure 1A, B). Fish need 1 to 2 hours to explore novel experimental surroundings [28,29]. During that time, exploration is an activity of highest priority [30,31] and could override parasite avoidance behaviour. That is why we analyzed the first and second 90-min periods separately. No difference in the NFR in the tests with solitary fish *vs* groups of 5 fish was found for the first 90 minutes (Two-way ANOVA, *F*_1,1_ = 0.008; *P* = 0.929. LSD post-hoc test: *P* = 0.628). During the last 90 minutes, the NFR (parasitized compartment) for tests on fish in groups was significantly less than for tests on solitary fish (Two-way ANOVA, *F*_1,1_ = 2.83; *P* = 0.099. LSD post-hoc test: *P* = 0.027), indicating that fish in groups better avoided the parasitized compartment in the second half of the test. Another way to compare grouped and solitary fish was to correlate the NFR for the parasitized compartment with elapsed time. No significant correlation was obtained for solitary fish (Spearman Rank Correlation: *R*_*S*_ = -0.325, *P* = 0.303), while there was highly significant negative correlation for fish in groups (*R*_*S*_ = -0.972, *P* < 0.0001). In fact, in tests with grouped fish, not a single fish was observed in the parasitized compartment after 150 minutes (Figure 1B).

### Experiment 2

**Figure 2:**
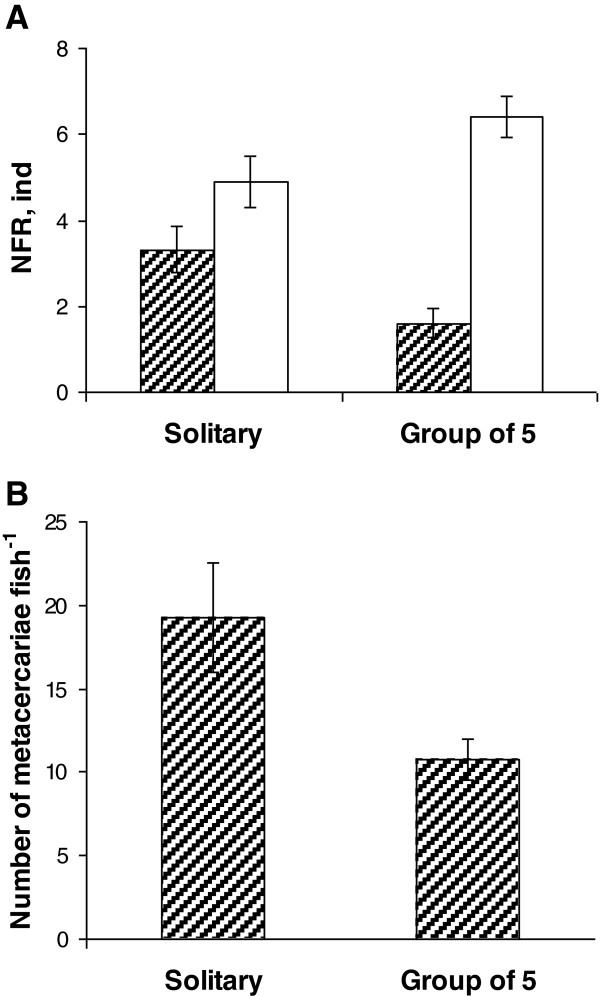
**Distribution of fish between two compartments and abundance of *Diplostomum pseudospathaceum *in Experiment 2. A** – “number of fish recorded”, NFR, pooled for 30 min, for solitary fish and fish in groups. Hatched bars: parasitized compartment; open bars: opposite, nonparasitized, compartment. **B** - abundance of parasites in solitary fish and fish in groups. Means and SE bars are shown.

Similar to Experiment 1, more *O. mykiss* were recorded in the nonparasitized compartment. Lower values of NFR in tests with both solitary fish and fish in groups were obtained for the compartment with parasites (Figure 2A) than for the opposite compartment (Two-way ANOVA, *F*_1,1_ = 103; *P* < 0.0001. LSD post-hoc test: *P* = 0.0001 for solitary fish, *P* < 0.0001 for fish in groups). The NFR values for the parasitized compartment pooled for the whole 30 min period were significantly lower in tests with groups of 5 fish than in tests with solitary fish (LSD post-hoc test: *P* = 0.002), which is in accordance with the results of the second half of Experiment 1 and with the idea of better avoidance of parasites by fish in groups.

Prevalence of infection was lower among fish in groups (66%) than in solitary fish (92%). Mean number of acquired metacercariae per fish was significantly lower when fish were in groups (mean ± SE: 10.8 ± 1.23 metacercariae per fish in grouped and 19.3 ± 3.27 in solitary fish) (Figure 2B) (Mann–Whitney U test: *Z* = 2.072; *P* = 0.038). Between-individual variation in the number of parasites per fish was noticeably higher across all fish tested in groups (coefficient of variation = 129%) than across all solitary fish (coefficient of variation = 81%).

**Figure 3:**
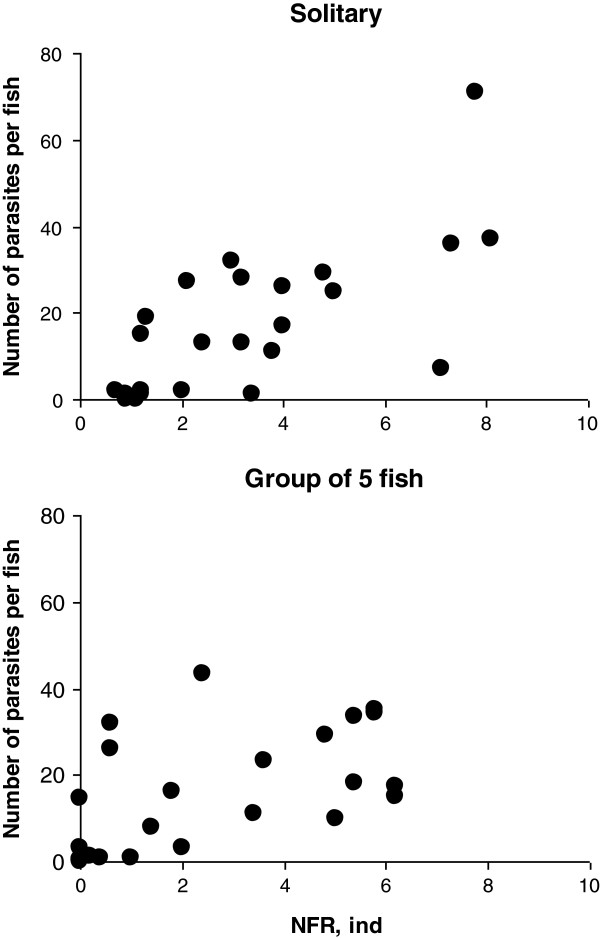
**Relationship between the number of acquired *Diplostomum pseudospathaceum *metacercariae and the “number of fish recorded”, NFR, in the compartment with parasites.** Upper panel: solitary fish. Lower panel: fish in groups. NFR, ind (pooled for 30 min) and the number of parasites are given per capita.

**Figure 4:**
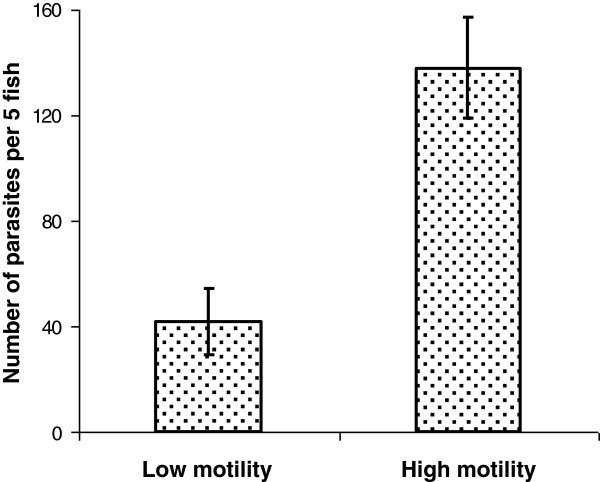
**Influence of host motility on the number of acquired *Diplostomum pseudospathaceum*.** Bars: means (± SE) number of *Diplostomum pseudospathaceum* parasites per 5 fish in low-motility and high-motility groups of *Oncorhynchus mykiss*.

The number of parasites per fish was positively correlated with the NFR values for the compartment with parasites (Figure 3); significant correlations were obtained for both solitary fish (*R*_*S*_ = 0.71; *P* < 0.001) and fish in groups (*R*_*S*_ = 0.66; *P* < 0.001). The relationship between the number of parasites per fish and the NFR for the parasitized compartment (ANCOVA: *F* = 29.00, *P* < 0.0001) was similar in tests on fish in groups and solitary fish (*F* = 0.14, *P* = 0.714). In the “high-motility” groups, mean number of acquired parasites was more than 3 times higher than that in the “low motility” groups (Figure 4) (Mann–Whitney U test: *Z* = -3.25; *P* = 0.001).

Fish in groups and solitary fish were exposed to the same concentration of cercariae (240 cercariae l^-1^). By the end of exposure in Experiment 2, the total number of parasites acquired by all fish did not exceed 0.5% of the initial concentration in the water, even in the trials with fish groups. Such a small decrease in parasite concentration in the environment indicates the conditions of a non-depleted patch. Despite the different number of fish exposed, the estimated difference in cercariae concentration in “group” and “solitary” trials at the end of the exposure was only a negligible 0.3%.

## Discussion

The results of this study show that 1) fish can avoid parasitized habitats, and 2) fish in groups avoid parasitized habitats better than solitary fish. It might be assumed that grouped fish are more attractive for cercariae due to stronger stimuli (e.g. odour) released by fish in groups that could be used by parasites to locate hosts. However, our results indicate the opposite. Fish grouping behaviour reduces the risk of parasitism in environments where parasites are unevenly distributed. The results suggest that infected fish in groups, displaying an abnormal behaviour, transfer information about parasites. Other fish in the group could use this information and avoid a risky patch (the parasitized compartment of the experimental tank) better than solitary fish with no opportunity to observe conspecifics. This resulted in lower mean intensity of infection within the grouped fish compared to solitary fish in the present experiment. This effect could not be attributed to the different number of fish exposed (“dilution effect”). It has earlier been shown that the cercarial density, rather than fish density, affects parasite transmission to fish [32]; see also the calculation in the last paragraph of Results.

Between-individual variation in the number of acquired metacercariae was pronouncedly higher in the 'group’ treatment (across all fish tested) than in the 'solitary’ treatment. Grouped fish not only acquired fewer parasites than solitary fish, but some of the group members did not get any parasites. Within a group, most of the acquired parasites were aggregated in a few fish individuals. The most common explanation for uneven distribution of parasites among hosts is differences in innate resistance of individual fish to parasites [8,33]. Our results provide new insight into this phenomenon. If differences in resistance would explain the individual differences in parasite intensity, then the between-individual variation should be more or less equal in experiments with solitary fish and fish in groups. We suggest that high variation in groups is related not only to individual differences in behaviour and physiology of fish, but also to information exchange within a group. Individuals within a group of fish are known to exchange information efficiently (reviewed in [11,17]). Better avoidance of the parasitized compartments by fish in groups indicates that information about risky sites is somehow spread within the group.

How can the fish attacked by *D. pseudospathaceum* cercariae signal to the others about the danger? Abundance of infection was much higher in those fish groups which moved more actively. Higher motility *per se* could result in more frequent visits to the parasitized compartment in our experiment. In addition, higher motility can increase ventilation volume which, in turn, may facilitate transportation of *D. pseudospathaceum* cercariae to fish [34]. Increased and conspicuous motility of fish injured by penetrating cercariae [13] and/or release of alarm substances [14] could be efficient signals. The most explorative fish would take a higher risk of infection while acquiring information about predators, food and surroundings. Less active fish within a group would benefit from avoiding risky activity and compensate for the information deficit by acquiring it from their more active conspecifics. As far as we know, this is the first empirical evidence suggesting transfer of information about infection risk within fish groups. Our results emphasize the role of behaviour as a factor affecting the between-individual variation in the number of acquired parasites.

Accumulation of parasites in a few fish could benefit the other members of a group, who would get a substantially lower infection or even avoid infection completely. A trade-off between faster exploration of a novel habitat and more cautious behaviour minimizing risks may contribute to within-group variability in acquired infection. Such a trade-off may depend on the “personality” of a host [35-37]. Another behaviourally-based mechanism stimulating aggregation of parasites on some host individuals was suggested by Poulin and co-authors [38]. They found that prior infection increased the probability that a fish would acquire further parasites during a subsequent exposure. The authors suggested that this may be due to parasite-induced behavioural changes. If the individual behavioural differences rendering some fish more vulnerable to parasites are consistent, then this could contribute to the highly aggregated distribution of parasites among host individuals. Aggregated distributions are a common feature of most fish parasites, including *D. spathaceum*[39]. The distribution of *D. spathaceum* is especially clumped in shoaling fish like *Coregonus lavaretus, Osmerus eperlanus* and *Rutilis rutilus*[39].

Avoidance of the parasitized compartment was much more pronounced in Experiment 2 where fish were allowed to explore the tank for 2 h prior to the tests. This time was required for habituation of fish to novel experimental conditions [29]. During the acclimation period, fish habituate and explore novel surroundings [28,40]. Exploration is an activity of high priority and could conflict with other vital activities [30,31]. In Experiment 1, where fish were allowed to explore the experimental arena for only 15 minutes prior to testing, acclimation and exploration may have brought fish often into the parasitized compartment. The effect of pre-test exploration was more pronounced in fish groups than in solitary fish. This indicates more efficient functioning of fish in shoals while performing exploration of a novel habitat see also [11,17].

The role of grouping behaviour of fish as a parasite-avoidance mechanism is much less studied than its role in anti-predator and foraging behaviour [11,18]. Nevertheless, infection-associated changes in shoaling behaviour [41], avoidance of heavily infected fish within a group [42], and avoidance of risky microhabitats with large and easily recognizable parasites like crustacean ectoparasites [12] have received attention. Using the *D. pseudospathaceum* – *O. mykiss* model, we have shown that grouping also substantially facilitates avoidance of much smaller parasites like the tiny cercariae of trematodes.

In the present experiments, fish in groups acquired fewer parasites, so that almost half (44%) of the fish in groups were uninfected. By contrast, only 8% of the 'solitary’ fish were free of parasites. Highly uneven distribution of acquired parasites across the fish in groups may influence transmission of *D. pseudospathaceum* at the next step of its life cycle when the parasite is involved in food webs [43]. Grouping seems to provide an efficient way to reduce intensity of parasitism for many members of the fish group. Thus, to minimize the risk of infection, fish may use not only costly physiological mechanisms, but also behavioural ones [44]. Our results suggest that avoidance of high-parasite-risk habitats can be an important factor contributing to the evolution and maintenance of group behaviour in animals.

## Conclusion

Our results show that fish can recognize parasitized areas and avoid them. This is especially important in heterogeneous habitats with patchily distributed, hard–to-detect parasites, like the cercariae of *D. pseudospathaceum* suspended in the water. Fish in groups recognize and avoid parasitized areas better than solitary fish. Fish in groups benefit from information about parasites in the environment gained from other members of a group. Grouped fish not only acquired fewer parasites than solitary fish, but some of the group members did not get any parasites. This suggests that grouping behaviour is an important mechanism of avoiding not only predators but also parasites. Fish use grouping, as well as individual behaviour and immune responses, to reduce the risk of parasitism. Variation in the number of parasites was much higher among fish in groups than among individually infected fish. We suggest that high variation in groups is related not only to individual differences in behaviour and physiology, but also to information exchange between members of a group. The most explorative fish would take a higher risk of infection while acquiring information about predators, food and surroundings. Less active fish within a group would benefit from avoiding risky activity and compensate for the lack of information by acquiring it from their more active conspecifics. If the individual differences in behaviour are consistent, this could contribute to increased variation in the number of parasites among host individuals. Our results support the idea that parasitism may be an important factor contributing to the evolution and maintenance of group behaviour in fish.

## Competing interests

The authors declare that they have no competing interests.

## Author’s contributions

VM and AP conceived the study, performed the experiments, and wrote the manuscript. JT advised on the statistical analysis and clarified the manuscript. ETV conceived and supervised the study. All authors read and approved the final manuscript.
